# Supplementary material for: Learning curve and surgical outcome of robotic assisted colorectal surgery with ERAS program
Source: Sci Rep. 2022 Nov 29;12:20566. doi: 10.1038/s41598-022-24665-w (PMC9709162; doi:10.1038/s41598-022-24665-w)
Supplement: Supplementary file 2 — Supplementary Information 2. [file 41598_2022_24665_MOESM2_ESM.zip › RAL Raw data-Ñ[▒K/124/HS_ñw╝╨░O▒Kñσ.pdf]

正式摘要(電子簽章：0000002929255524)		
W52 / W52 ☐英文標題(注意:無英文姓名-請補病人基本檔!)	☐【讀取】憑證	

臺中榮民總醫院
出院病歷摘要

轉入號： 轉出號：

[redacted]

**(8)**流水編號	
**(9)**入院日期	110/11/03 (2021) 直腸外科 W52 - 092
**(10)**轉科日期	
**(11)**出院日期	110/11/07 (2021) 住院天數計 4 日

診斷

- 入院:1. S-colon adenocarcinoma, FAV 35cm
- 出院:1. S-colon adenocarcinoma, FAV 35cm, s/p Robotic assisted AR on 2021/11/04

主訴

- Bloody stool noticed on 2021/10/10

病史

- This is a 38 years old female patient denied underlying disease. According to the patient, she had bloody stool on 2021/10/10. Then she went to CSMUH for help, CT and LGI scopy were done, showed FAV:35cm tumor, s/p biopsy, which pathology reported adenocarcinoma. Under the impression of S-colon cancer, she visit our CRS OPD for second opinion. Repeat colonoscopy and tatto orientation was done on 2021/11/03. Then, she was admitted to our ward for operation management.

體檢發現

-
  - General appearance :
    - easy looking , conscious clear , without cardiopulmonary distress
    - Vital signs自動帶入date 體溫(BT) 脈搏(P) 呼吸(R) 血壓(BP) 身高(BH) 體重(BW) BMI
      20211103 36.4℃ 94次/分 18次/分 120/68mmHg 160.1cm 75.9kg 29.6
    - Vital signs: B.P. 120/68 mmHg , P.R. 94 /min , R.R. 18 /min , B.T. 36.4 ℃
  - HEENT :
    - Eyes: no anemic conjunctiva, no icteric sclera, normal EOM
    - ENT: no otorrhea, no rhinorrhea, no erythematous mucosa, no injected tonsil, no deformity
  - Neck: supple, freely movable, trachea midline,no deformity, no engorged jugular vein , no palpable thyroid or lymph node enlargement
  - Chest:
    - Inspection: Grossly normal, symmetric and fully expansion, no accessory muscle used, no operation scar, no spider angiomata, no gynecomastia
    - Palpation: no palpable mass or nodule, no subcutaneous emphysema, no tenderness
    - Ausculation: clear breathing sound
  - Heart:
    - Palpation: no thrill, no heave
    - Percussion: normal heart size
    - Ausculation: regular heart beat, normal S1, S2, no gallop, no murmur
  - Extremities: No deformity, full ranged of motion without limitation, no clubbing finger or nail cyanosis, no pitting edema, no ulcer or pigmentation, no obvious skin lesion, no varicose vein, no bone, joint or muscle tenderness

CRS CONDITION

- Karnofsky performance status: (100) Normal, no complaints, no evidence of disease
- PE of Abdomen:

- - CEA: . ng/ml
  - Imaging Studies:
  - Colonoscope report: Adenocarcinoma, FAV 35cm
  - Figures:
    1. colon & rectum

手術日期及方法

- 手術日期及方法20211104 1. Robotic (XI) assisted anterior resection
  2. Intraoperative colonoscopy : (-).
  3. High ligation (+) : At IMA root. Left colic artery and IMV were also ligated via robotic endoclips.
  4. Take down from the splenic flexure : (-)
  5. Robotic endoGIA (45-4.3) X1 + EEA (29mm) were applied
  6. Proximal cut end: 8 cm, distal cut end: 5 cm
  7. ADHESION BARRIER FILM was placed below the central abdominal wound

住院治療經過

- After admission, she received colonoscopy with tatto localization on 11/03. Robotic assisted AR was done on 2021/11/04. She start Bland I diet on 11/05 and Bland II diet on 11/06, tolerate well. Under relative stable condition, she was discharged on 2021/11/07, OPD f/u.

合併症

一般檢查

- 累積報告 :

DATE	TIME	ALB	TP	BIL.T	BIL.D	ALKP	AST	ALT	LDH	AMY	LIPASE	NH3	Ferritin	UA
1101104	1432			0.67			15							

DATE	TIME	WBC	RBC	HGB	HCT	MCV	PLT	BLST	NEUT	BAND	SEG	LYM	MON	EOS	BAS
1101104	1432	15760		12.4	38.1		282		93.8			5.1	0.8	0.1	0.2

DATE	TIME	NA	K	CL	BUN	CREAT	eGFR	CA	Mg	P	CRP	ESR	PCT	D-Dimer	LACTATE
1101104	1432		4.1		5	0.69	101.2								
1101104	1433														22.6

特殊檢查

病理報告

其它

出院時情況

- 3.改門診治療

出院指示

-

藥名	劑量	劑量單位	用法	頻次	數量	數量單位	現況	天數	單位
(口)Takepron OD tab 30mg	1	TAB	PO	QDAC	3	TAB	TH	3	天
Mag. Oxide tab	2	TAB	PO	TID	18	TAB	TH	3	天
Nincort oral geL 6gm		TUB	TOP	TID	0	TUB	TH	1	瓶
Ultracet tab	1	TAB	PO	Q6H	12	TAB	TH	3	天

出院計劃

- 門診日期
  - date on 2021/11/10
- OPD f/u

主治醫師 2062F 蔣鋒帆 / 住院醫師 2339C 蔡濬樟

正式摘要(電子簽章：**000000229255524)**

W52 / W52

doctorRank:B
